# Identification and verification of communicating accessory bile duct associated with a biliary circuit by modified and dynamic intraoperative cholangiography during laparoscopic cholecystectomy

**DOI:** 10.1259/bjrcr.20230037

**Published:** 2023-07-03

**Authors:** Fumio Chikamori, Ryo Yamada, Koji Ueta, Sunao Uemura, Kazuhisa Onishi, Mitsuteru Yoshida, Nobuyuki Tanida, Hiromichi Yamai, Hisashi Matsuoka, Norihiro Hokimoto, Jun Iwabu, Kai Mizobuchi, Akira Marui, Niranjan Sharma

**Affiliations:** 1 Department of Surgery, Japanese Red Cross Kochi Hospital, Hadaminamimachi, Kochi, Japan; 2 Adv Train Gastroint & Organ Transp Surgery, Dunedin, New Zealand

## Abstract

Communicating accessory bile duct (CABD) is a rare anatomical anomaly of the bile duct and forms a biliary circuit. It is difficult to identify during laparoscopic cholecystectomy (LC) without the use of intraoperative cholangiography (IOC). A modified IOC, in which tube insertion was performed through the infundibulum of the gallbladder, was evaluated dynamically. This procedure allowed us to accurately identify and verify the presence of CABD, a biliary circuit, and the short cystic duct. The short cystic duct could be separated safely without damaging the biliary circuit. Modified and dynamic IOC is recommended for identifying and verifying the presence of CABD during LC.

## Introduction

Communicating accessory bile duct (CABD) associated with a biliary circuit is a rare biliary anomaly.^1–4^ CABD could be demonstrated by magnetic resonance cholangiopancreatography (MRCP),^5^ but its pre-operative diagnostic rate has not been significantly high.^2^ Identifying CABD is very difficult during laparoscopic cholecystectomy (LC); without the use of intraoperative cholangiography (IOC). We report modified and dynamic IOC,^6^ which enabled accurate imaging of the CABD, biliary circuit, and short cystic duct during LC in a case.

## Case

A 65-year-old female came to the hospital complaining of upper abdominal pain. Physical examination on admission revealed tenderness in the right abdomen and a body temperature of 37.0°C. Laboratory results were WBC 23500 /µl (normal level: 3500–8000), total bilirubin 3.2 mg dl^−1^ (0.3–1.3), ALT 9 U l^−1^ (5-27), AST 20 U l^−1^ (10-32), ALP 82 U l^−1^ (109-344),γ-GTP 37 U l^−1^ (8-45), procalcitonin 1.35 ng ml^−1^ (0–0.49) and CRP 21.55 mg dl^−1^ (<0.16). Based on these results, acute cholecystitis was suspected.

**Figure 1.**
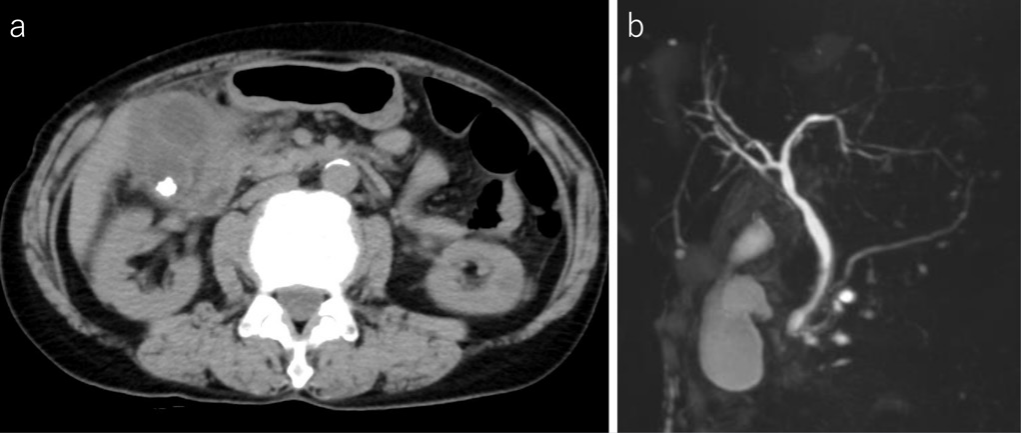
Abdominal plain CT (**a**) and MRCP (**b**) on initial admission. Plain CT (**a**) showed multiple stones, swelling, and wall thickening of the gallbladder. The anteroposterior view of MRCP (**b**) does not clearly show a biliary circuit and cystic duct (arrow). MRCP, magnetic resonance cholangiopancreatography.

Ultrasound, plain CT, and MRCP showed a swollen, thick-walled gallbladder with multiple stones (Figure 1a). A small amount of fluid was noted around the gallbladder. Biliary circuit and cystic duct were obscured (Figure 1b). Diagnosis of acute cholecystitis with an abscess around the gallbladder was made. Percutaneous transhepatic gallbladder drainage (PTGBD) was performed. Bile culture showed presence of *Enterobacter cloacae*.

**Figure 2.**
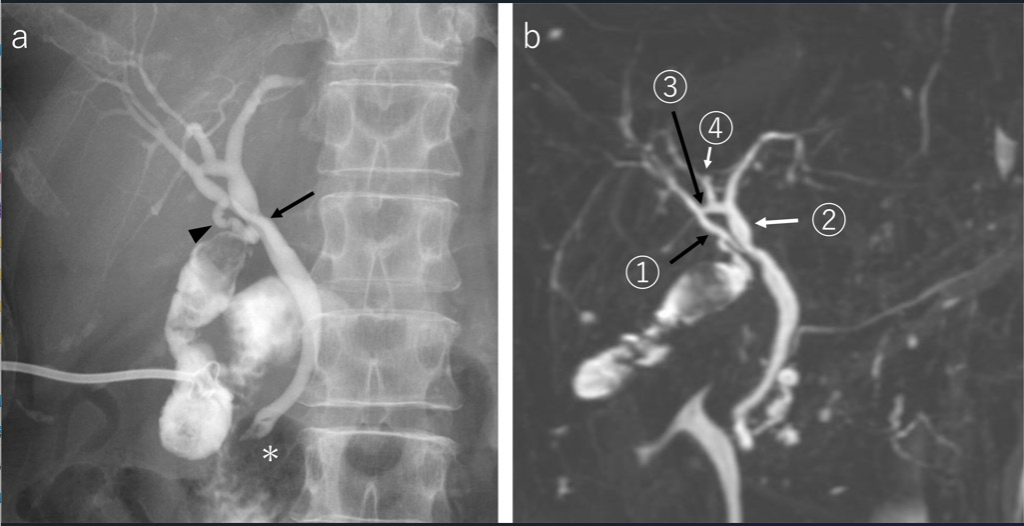
Cholangiogram via PTGBD tube (**a**) and MRCP (**b**) before surgery. Cholangiogram via PTGBD tube (**a**) shows that the RPHD drains into the LHD. The cystic duct (arrowhead) running appears normal due to the density of the contrast medium (arrow). The presence of a biliary circuit (arrow 1–4) is suspected on the reexamination of MRCP just before the third hospitalization (**b**). Arrow 1 indicates RAHD. Arrow 2 indicates LHD. Arrow 3 indicates CABD. Arrow 4 indicates RPHD. CABD, communicating accessory bile duct; LHD, left hepatic duct; MRCP, magnetic resonance cholangiopancreatography; PTGBD, percutaneous transhepatic gallbladder drainage; RAHD, right anterior hepatic duct; RPHD, right posterior hepatic duct.

The cholangiography via PTGBD tube revealed common bile duct (CBD) stones. We could show that the right posterior hepatic duct (RPHD) communicated with the left hepatic duct (LHD). However, the contrast misidentified the cystic duct as joining the common hepatic duct (CHD) (Figure 2a). Due to the intense focus on the presence of stones in CBD, the presence of CABD was overlooked. The patient was discharged 7 days after PTGBD. She was readmitted and treated by endoscopic sphincterotomy for CBD stones 20 days after PTGBD. The presence of CABD was missed even at the time of the procedure. At the patient’s request, the PTGBD tube was removed 4 weeks after PTGBD. An interval surgery was scheduled 4 months later. The presence of a biliary circuit was suspected on the re-examination of MRCP just before the third hospitalization (Figure 2b), but the length of the cystic duct was considered to be sufficient by the previous cholangiogram via the PTGBD tube (Figure 2a).

**Figure 3.**
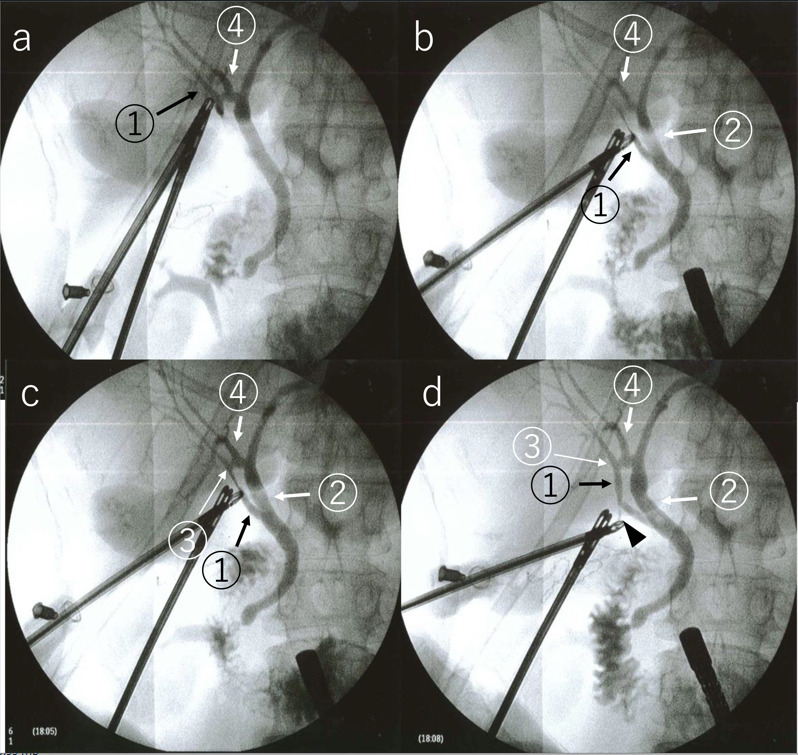
Modified and dynamic IOC during LC The first cholangiogram (**a**) shows the RAHD (arrow 1), RPHD (arrow 4), LHD (arrow 2), CHD, and CBD in that order, but a biliary circuit is not visualized. After pulling the cholangiograsper caudally, the second cholangiogram (**b**) shows the caudal side of the RAHD (arrow 1). Further injection of contrast medium (**c**) shows communication (arrow 3) with the RPHD (arrow 4). After pulling the cholangiograsper more caudally, the third cholangiogram (**d**) shows all biliary structures: the RAHD (arrow 1), CABD (arrow 3), biliary circuit (arrow 1–4), and short cystic duct (arrowhead). Arrow 1 indicates RAHD. Arrow 2 indicates LHD. Arrow 3 indicates CABD. Arrow 4 indicates RPHD. CABD, communicating accessory bile duct; IOC, intraoperative cholangiography; LC, laparoscopic cholecystectomy; LHD, left hepatic duct; MRCP, magnetic resonance cholangiopancreatography; PTGBD, percutaneous transhepatic gallbladder drainage; RAHD, right anterior hepatic duct; RPHD, right posterior hepatic duct.

**Figure 4.**
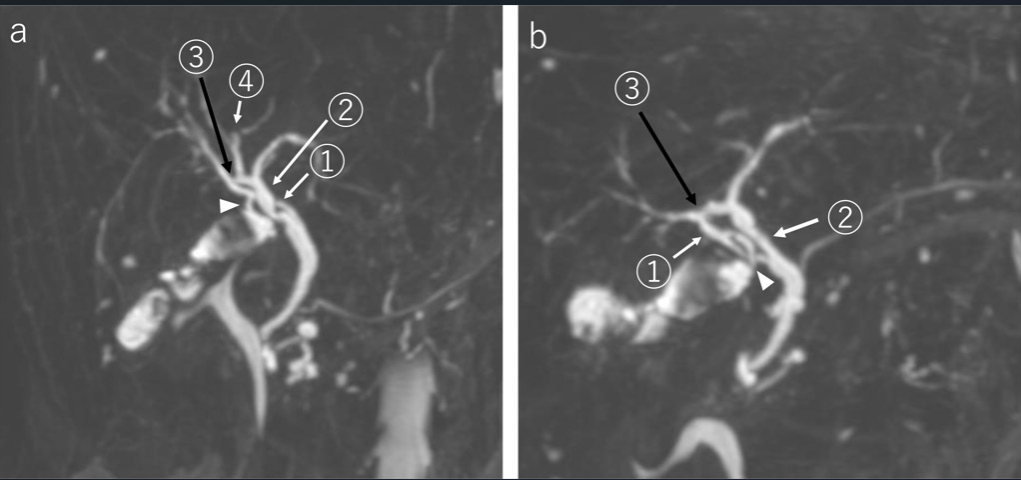
Cranial view (**a**) and caudal view (**b**) of MRCP before surgery. The cranial view (**a**) of MRCP shows the RAHD (arrow 1) and cystic duct (arrowhead). Caudal view (**b**) of MRCP shows the RAHD (arrow 1), cystic duct (arrowhead), and biliary circuit. Arrow 1 indicates RAHD. Arrow 2 indicates LHD. Arrow 3 indicates CABD. Arrow 4 indicates RPHD. CABD, communicating accessory bile duct; LHD, left hepatic duct; MRCP, magnetic resonance cholangiopancreatography; RAHD, right anterior hepatic duct; RPHD, right posterior hepatic duct.

During LC, mild inflammation of the gallbladder was observed. A modified and dynamic IOC was performed in the same manner as previously reported.^6^ Cannulation was performed from the infundibulum after exposure of the gallbladder neck. Initial IOC showed right anterior hepatic duct (RAHD), RPHD, LHD, CHD, and CBD in that order, but a biliary circuit was not visualized (Figure 3a). As the biliary circuit was not visualized in the first instance; the second IOC was performed slightly pulling the cholangiography forceps caudally. By doing so, the contrast showed the caudal side of the RAHD (Figure 3b). Further injection of contrast medium showed communication with the RPHD (Figure 3c). The final IOC after more caudal traction of the cholangiography forceps showed all biliary structures: the RAHD, CABD, biliary circuit, and short cystic duct (Figure 3d). Then, we reviewed the MRCP again and reflected on the insufficient pre-operative interpretation (Figure 4a and b). The short cystic duct was carefully separated after clipping. The post-operative period was uneventful and the patient was discharged on the fourth day after LC. On MRCP 2 weeks after LC, the biliary circuit was clear and was intact.

## Discussion

This is an educational case report of CABD with a biliary circuit that was verified by a modified and dynamic IOC^6^ during LC. Insufficient identification of cystic duct is one of the common causes of bile duct injuries. Some authors warned about the presence of branching variation in the right hepatic duct^7–9^: the RPHD connected to the LHD in 9–17% of cases, and the RPHD directly connected to the CHD in 3–18%. Benson et al^10^ reported the variations of the cystic duct: the presence of an accessory hepatic duct in 1.4% of cases, the cystic duct entering the right hepatic duct in 0.7%, and the cholecystohepatic duct in 0.7%.

The presence of CABD associated with a biliary circuit is a rare bile duct anomaly. CABD has also been reported as interhepatic duct or double hepatic duct.^11–14^ Yamamoto et al^15^ reported that the intrahepatic bile ducts at the hilum have many irregular side branches which may communicate with each other. The development of such communications seems to develop into CABD. Some authors cautioned against biliary circuit damage in patients with CABD.^2–4^

MRCP is an essential examination for pre-operative biliary evaluation.^5,16^ However, in some occasions, visualization of the cystic duct–hepatic duct junction becomes poor in acute cholecystitis as in this case (Figure 1B). Early surgery is currently recommended for acute cholecystitis,^17^ but the presence of this anomaly should be recognized. In general, accessory bile duct ligation is less likely to cause adverse events.^5,16^ However, CABD-associated biliary circuit injury in LC was reported.^4^ In clinical practice, CABD associated with a biliary circuit is not widely known to surgeons. Thus, it is necessary to pay attention to part 1 in Figure 2B in LC. Using modified and dynamic IOC, the initial pre-operative misinterpreted orientation of the cystic duct was accurately diagnosed in this case.

Modified dynamic IOC is recommended when bile duct anomalies are suspected on MRCP or the bile duct anatomy is unclear during difficult cholecystectomy. In this case, if MRCP had been interpreted in detail, a definitive diagnosis could have been made pre-operatively. Even with a pre-operative diagnosis of CAHD with a biliary circuit, modified and dynamic IOC immediately prior to cystic duct separation would be useful to avoid biliary circuit injury. As with aircraft accident countermeasures, it is important to take multiple measures to prevent bile duct injury. If the bile ducts overlap is severe, the C-arm should be rotated for oblique viewing.^6^ In this case, only the frontal view of the modified and dynamic IOC showed a clear view of the biliary circuit, so oblique imaging was not necessary.

Biliary injury can be avoided by the modified IOC without a cystic duct incision. Dynamic cholangiography with moving the tip of the forceps compensates for the low resolution of IOC. There have been no reports of CABD with a biliary circuit verified by modified and dynamic IOC. This case could serve as a reminder that thorough anatomical confirmation is necessary before or at the time of cholecystectomy.

## Learning points

1. Knowledge of CABD with a biliary circuit is important in clinical practice.
2. Exhaustive imaging with MRCP and modified dynamic IOC provides an accurate diagnosis of CABD with a biliary circuit.
3. Because the modified and dynamic IOC allows us to accurately confirm CABD, a biliary circuit, and the short cystic duct, the cystic duct can be separated safely without damage to the biliary circuit in LC.
